# The diagnostic potential of urine in paediatric patients undergoing initial treatment for tuberculous meningitis

**DOI:** 10.1038/s41598-024-70419-1

**Published:** 2024-08-22

**Authors:** Simon Isaiah, Johan A. Westerhuis, Du Toit Loots, Regan Solomons, Marceline Tutu van Furth, Sabine van Elsland, Martijn van der Kuip, Shayne Mason

**Affiliations:** 1 Human Metabolomics, Faculty of Natural and Agricultural Sciences, North‒West University, Potchefstroom, South Africa; 2 Biosystems Data Analysis, Swammerdam Institute for Life Sciences, University of Amsterdam, Amsterdam, The Netherlands; 3 Department of Paediatrics and Child Health, Faculty of Medicine and Health Sciences, Stellenbosch University, Cape Town, South Africa; 4 Pediatric Infectious Diseases and Immunology, Vrije Universiteit, Amsterdam University Medical Centers, Emma Children’s Hospital, De Boelelaan 1117, Amsterdam, The Netherlands; 5 MRC Centre for Global Infectious Disease Analysis, Imperial College London, London, UK

**Keywords:** Diagnostic, Tuberculous meningitis (TBM), Proton magnetic resonance (^1^H-NMR), Urine, Pediatric, Metabolomics, Biochemistry, Metabolomics, Diseases, Diagnostic markers

## Abstract

Tuberculous meningitis (TBM)—the extrapulmonary form of tuberculosis, is the most severe complication associated with tuberculosis, particularly in infants and children. The gold standard for the diagnosis of TBM requires cerebrospinal fluid (CSF) through lumbar puncture—an invasive sample collection method, and currently available CSF assays are often not sufficient for a definitive TBM diagnosis. Urine is metabolite-rich and relatively unexplored in terms of its potential to diagnose neuroinfectious diseases. We used an untargeted proton magnetic resonance (^1^H-NMR) metabolomics approach to compare the urine from 32 patients with TBM (stratified into stages 1, 2 and 3) against that from 39 controls in a South African paediatric cohort. Significant spectral bins had to satisfy three of our four strict cut-off quantitative statistical criteria. Five significant biological metabolites were identified—1-methylnicotinamide, 3-hydroxyisovaleric acid, 5-aminolevulinic acid, N-acetylglutamine and methanol—which had no correlation with medication metabolites. ROC analysis revealed that methanol lacked diagnostic sensitivity, but the other four metabolites showed good diagnostic potential. Furthermore, we compared mild (stage 1) TBM and severe (stages 2 and 3) TBM, and our multivariate metabolic model could successfully classify severe but not mild TBM. Our results show that urine can potentially be used to diagnose severe TBM.

## Introduction

Tuberculous meningitis (TBM) is a devastating and severe form of extrapulmonary tuberculosis (TB) that affects the central nervous system, especially the meninges that surround the brain^1,2^. TBM causes substantial mortality and morbidity worldwide, with sub-Saharan Africa being by far the most severely affected region globally. Infants and children are the most vulnerable group to TBM, resulting in death or neurological deficits in those who are fortunate enough to survive^3–7^. In South Africa, the Western Cape Province has a high incidence of tuberculosis (TB) (681 per 100,000) in infants and children under the age of 14 years^8–10^. The diagnosis of TBM is a major clinical challenge since this insidious infection often masks nonspecific symptoms that overlap with a spectrum of neurological complications that mimic other forms of meningitis^11^. Despite the availability of effective treatments, TBM remains a severe disease in progressive stages with slow onset, particularly in resource-constrained settings. The vague clinical manifestations of TBM make the initial distinction of this inflammatory disease a formidable task, especially in the early stages^7^. A study showed an outcome difference between stages (mortality stage I: 1%, stage II: 10% and stage III: 34% with morbidity of approximately 27% stage I, 41% stage II and 71% stage III)^12^, and the lack of a definitive diagnostic gold standard in children makes the diagnosis even more complex^13^. One of the mainstays of TBM diagnosis is the detection of *Mycobacterium tuberculosis* (*M.tb*) in cerebrospinal fluid (CSF), which requires a lumbar puncture—an invasive procedure. Traditional diagnostic methods for TBM, such as microscopy with acid-fast staining and culture, are characterized by their limited sensitivity and the long time required to obtain results^7,11,14^. Polymerase chain reaction (PCR) is a valuable diagnostic tool; however, a meta-analysis of the commonly used Xpert MTB/RIF in TBM showed high accuracy but was unable to rule out TBM^15^. Similarly, Xpert MTB/RIF Ultra improves the sensitivity of Xpert MTB/RIF but is also unable to exclude TBM^16^. Currently, no single diagnostic test is sufficient for accurate differential diagnosis of TBM; hence, a combination of multiple diagnostic tests has been proposed.

Biomarkers can be used to diagnose, monitor, or predict the progression of a disease^17^. However, currently, there are no highly specific diagnostic biomarkers for TBM. Hence, it remains a priority to identify markers that allow timely and accurate diagnosis for optimal treatment outcomes. Efforts to overcome these diagnostic challenges in TBM continue to progress, and researchers are working to improve and refine diagnostic approaches to allow for the earlier identification and subsequent treatment of TBM patients. Urine-based diagnostic tests are becoming increasingly useful for diagnosing TB because they are noninvasive and easy to perform. Urinary TB lipoarabinomannan (TB-LAM) assay detects the presence of LAM, a cell wall protein of *M.tb*, especially in HIV-infected people with low CD4 counts, and Xpert MTB/RIF Ultra also detects the presence of *M.tb* DNA in urine^18–20^; however, the results of TB-LAM detection have been suboptimal, and there is a paucity of *M.tb* DNA in urine. Metabolomics is the study of the complete set of small molecule metabolites in a biological system^21^. Metabolites are the end products of metabolic pathways, and their levels can be affected by a variety of factors, including diseases^22^. From our urinary metabolomics studies on TBM, we described the biochemical characterization of the urinary profile of TBM^23^ and identified markers of dysbiosis^24^, paving the way for the use of urinary metabolomics for diagnostic purposes. In this investigation, we used ^1^H-NMR spectroscopy because it is a rapid, highly repeatable, and robust metabolomics analytical platform. By highlighting the diagnostic difficulties associated with TBM, this study aimed to identify biomarkers of TBM with high precision and specificity using noninvasively collected urine.

## Materials and methods

### Sampling

This study included infants and children (13 years or younger) from the Western Cape province of South Africa, a region with a high burden of TB, particularly in children^8,9,25,26^. Children with clinical symptoms and signs suggestive of meningitis who initially arrived at basic-level and regional health centres were referred to the Department of Paediatrics and Child Health at Tygerberg Hospital in the Western Cape Province of South Africa. According to the universal research case definition criteria for TBM^7^, all participants in our study (n = 32) were diagnosed with definite TBM and staged according to the revised British Medical Research Council (BMRC) criteria, which were used to classify TBM severity as follows: stage 1, Glasgow Coma Scale (GCS) of 15, without focal neurological deficit; stage 2, GCS of 15 with focal neurological deficit or GCS of 11–14 with or without focal neurological deficit; and stage 3, GCS < 11 with or without focal neurological deficit^27^. TBM diagnosis was based on the identification of *M.tb* in CSF by microscopy, culture, and/or commercial nucleic acid amplification tests. All patients with TBM were stabilized in the Tygerberg Hospital Paediatric Neurology ward and treated with a short-intensified anti-*M.tb* drug regimen that included high-dose rifampicin, isoniazid, pyrazinamide, and ethionamide given for 6 months if HIV uninfected and 9 months if HIV co-infected^28,29^. Urine samples from TBM patients were collected upon discharge from the hospital; the median time from admission to discharge in the study setting over the past 38 years was 16 days, the interquartile range was 12–23 days^24^. HIV co-infection considerably complicates an already complex metabolic profile; hence, participants with a positive or unknown HIV status were excluded from this investigation.

### Sample collection

This study compared the urine of a control group (n = 39) with that of an experimental group of paediatric patients (n = 32). The controls, anonymously collected with written and informed consent, were classified as ‘normal’ paediatric patients who were admitted to Tygerberg Hospital. Here, ‘normal’ is based on the following benchmark: paediatric patients without meningitis, without neurological symptoms, and from the same geographic location as the patients with TBM. No further clinical data on the controls were available due to ethical constraints. The group of patients with TBM was subdivided into stage 1 TBM (n = 8), stage 2 TBM (n = 10) and stage 3 TBM (n = 14). Clinical symptoms included headache, fever, nausea/vomiting, photophobia, meningeal irritation, and/or neck stiffness^9^. The first urine sample after hospital discharge was obtained and classified as time point 1 (T1). Table 1 provides a more detailed description of the demographics and clinical information of the TBM patients.

**Table 1:** Demographic and clinical characteristics of the TBM patients in this study stratified into stages 1, 2 and 3.

Criteria	Stage 1 TBM	Stage 2 TBM	Stage 3 TBM
	n (%)	n (%)	n (%)
Male gender	4 (50)	6 (60)	7 (50)
Age (months)*	65 [30–117]	45.5 [10–135]	42 [22–140]
Clinical symptoms			
Fever	4/8 (50)	4/10 (40)	12/14 (86)
Night sweats	0	1/10 (10)	2/14 (14)
Poor feeding	2/8 (25)	2/10 (20)	5/14 (36)
Weight loss	3/8 (36)	2/10 (20)	5/14 (36)
Vomiting without diarrhoea	3/8 (36)	1/10 (10)	4/14 (29)
Persistent (> 14 days) coughing	1/8 (13)	0	0
Headache	2/8 (25)	2/10 (20)	1/14 (7)
Seizures	3/8 (36)	1/10 (10)	5/14 (36)
Lethargy	2/8 (25)	1/10 (10)	2/14 (14)
Neurological signs			
GCS*	15 [15]	13 [12–15]	10 [7–15]
Meningism	0	4/10 (40)	3/14 (21)
Focal motor deficit	0	2/10 (20)	4/14 (29)
Cranial nerve palsy	0	1/10 (10)	3/14 (21)
Raised ICP	0	2/10 (20)	4/14 (29)
Neuroimaging (CT brain)			
Hydrocephalus	2/8 (25)	5/10 (50)	12/14 (86)
Infarctions	0	2/10 (20)	1/14 (7)
Tuberculoma	2/8 (25)	1/10 (10)	2/14 (14)
Meningeal enhancement	2/8 (25)	2/10 (20)	6/14 (43)
VP shunt present	0	1/10 (10)	4/14 (29)
Other radiology			
CXR (signs of pulmonary TB)	1/8 (13)	3/10 (30)	2/14 (14)
Laboratory values			
Blood sodium (mmol/L)*	139 [136.7–142.2]	136 [134.7–137.7]	131.5 [124.5–138]
Blood total protein (g/L)*	34 [17–51]	74 [73–75]	78.5 [76.7– 80.2]
Blood glucose (mmol/L)*	0	2.95 [1.47–4.42]	7.1 [6 –7.4]
Blood lipids*	0	0	1.95 [0.97–2.92]
CSF protein (g/L)*	0.53 [0.12–6.20]	0.93 [0.83–1.27]	0.84 [0.76–1.93]
CSF glucose (mmol/L)*	2.6 [1.27–3.6]	2.05 [1.2–2.71]	1.8 [0.8–2.85]
CSF lymphocytes (cells/µL)*	40 [20–60]	157 [103–410]	57 [40–83]
CSF lymphocytes (%)*	43 [21.7–65.2]	92 [88–94.8]	96 [94.5–100]

*CXR* chest radiography, *GCS* Glasgow coma scale, Sex (M = male, F = female), *CSF* cerebrospinal fluid, *ICP* intracranial pressure, *VP* Ventriculoperitoneal.

*Median with interquartile range.

### Sample transport, storage, and handling

The urine samples were stored at − 80 °C in a dedicated freezer at Stellenbosch University's Division of Molecular Biology and Human Genetics. These samples were then collectively transferred overnight—frozen on dry ice—to the BSL-3 Laboratory at Human Metabolomics North‒West University, Potchefstroom campus and stored at − 80 °C. On the day that the metabolomics analyses were performed, the samples were allowed to thaw in a biological safety cabinet. A pooled quality control (QC) urine sample was created by aliquoting 50 µL of each sample and pooling them into a single tube.

### Sample preparation and ^1^H NMR analysis

Prior to analysis, all urine samples were thawed to room temperature. To remove any particulates and macromolecules, 600 µL of urine was centrifuged at 12,000 g for 5 min. A 540 µL volume of supernatant was added to 60 µL of NMR buffer solution [1.5 M potassium phosphate solution in deuterium oxide with internal standard trimethylsilyl-2,2,3,3-tetradeuteropropionic acid (TSP); pH 7.4]. The sample was vortexed and centrifuged at 12,000 g for 5 min, and 540 µL of the supernatant was transferred to a 5 mm NMR glass tube and capped. All the samples were loaded in random order onto the NMR autosampler, with the QC samples periodically interspaced. The QC samples were used as a quality assurance method to monitor the analytical variation. The instrument used was a Bruker Avance III HD 500 MHz NMR spectrometer with a triple-resonance inverse (TXI) ^1^H (^15^N, ^13^C) probe head and x, y, and z gradient coils. The ^1^H spectra were captured as 128 transients at 32 K data points with a spectral width of 12,000 Hz. The sample temperature was kept constant at 300 K, and the H_2_O resonance was pre-saturated using single-frequency irradiation with a 4 s relaxation delay and an 8 μs excitation pulse. The deuterium signal was used to automatically shim the samples. The resonance line widths of half of the TSP peak were less than 1 Hz, indicating good resolution. Automatic Fourier transformation and phase and baseline correction were performed. The NMR data were processed using Bruker Topspin (V3.5). Bruker AMIX (V3.9.14) was used for identification and quantification^30^.

### Data pre-processing

The ^1^H NMR spectral output was binned at set widths of 0.02 ppm, and probabilistic quotient normalization^31^ was applied to account for dilution differences in the urine samples. A data matrix was constructed, with bins in columns and samples in rows and data entries as the spectral data intensity. The bins affected by the suppressed water peak at ~ 4.72 ppm were removed. The coefficient of variation (CV) was checked in all bins for the QC samples, and any bin with a QC CV > 10% was removed. Therefore, all remaining bins were considered reliable (< 10% analytical variation) for biological interpretation.

### Statistical analysis

**Fig. 1:**
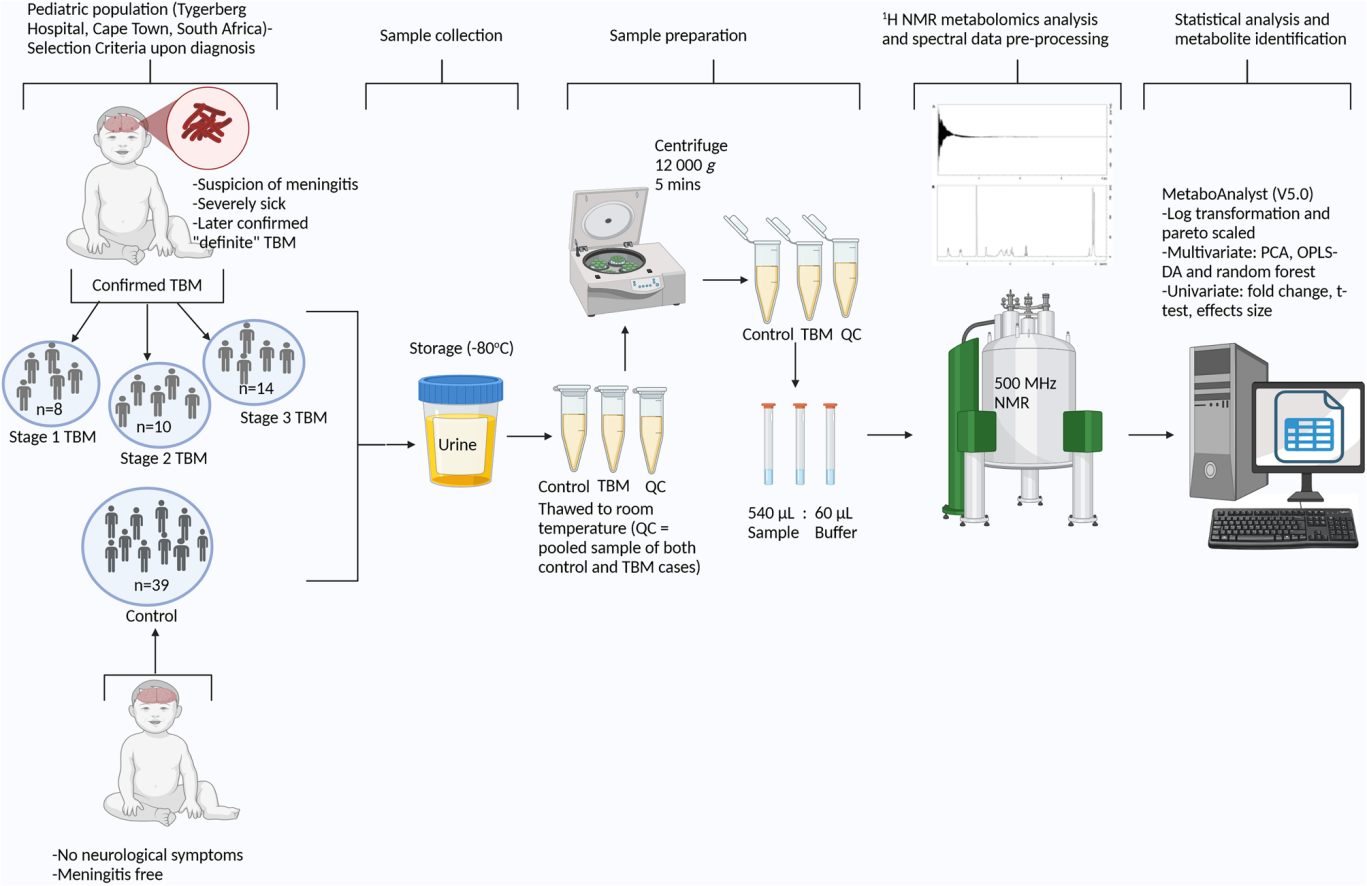
Schematic study design illustrating the experimental workflow – from (left to right): sampling, sample collection, sample preparation, 1H-NMR analysis, and statistical analysis.

All the statistical analyses were performed with Microsoft Excel, MetaboAnalyst 5.0, an online metabolomics tool (www.metaboanalyst.ca/), and GraphPad Prism 10. The data were log transformed and Pareto scaled for all multivariate analyses [principal component analysis (PCA), orthogonal partial least squares discriminant analysis (OPLSDA), and random forest (RF) alongside OPLSDA to support the results (discriminatory variables were compared)], while all univariate statistics were analysed using nonparametric tests. Unsupervised PCA was used to obtain a visual overview of the data—qualitative assessment of group differentiation and/or clustering. Subsequently, four quantitative statistical tests were performed with defined and strict critical cut-off criteria. A spectral bin was considered important for further investigation of its diagnostic potential if it met at least three of the following four criteria: 1) OPLSDA with a VIP > 1.5 (supported by an RF out-of-the-bag (OOB) value < 0.05), 2) a Wilcoxon t test with an FDR p value < 0.01, 3) a fold change > 5.0, or 4) Hedge’s effect size g value > 1.0. The metabolites within the important bins were identified based on 1D and 2D ^1^H-NMR pure compound spectral libraries, and the metabolite concentrations were calculated as mmol/mol creatinine. Subsequently, violin plots were used to illustrate the distribution of the frequency of the concentrations of these important metabolites, as well as their medians, interquartile ranges, and statistical significance (p < 0.01, with multiple test corrections). Receiver operating characteristic (ROC) curves of the most important biological variables were generated to assess their diagnostic potential, and correlation analysis was performed to assess whether the biological diagnostic markers and unknowns had any strong correlations (r > 0.6), with statistical significance (p < 0.05) with the metabolites of the medication. Figure 1 illustrates the overall experimental design.

### Ethics approval and consent to participate

All research was performed in accordance with relevant guidelines/regulations, and informed consent was obtained from all participants and/or their legal guardians. All research was performed in accordance with the Declaration of Helsinki. Ethical approval was obtained from the Stellenbosch University Health Research Ethics Committee (HREC) (ethics approval number: N16/11/142 and N11/03/061 for TBM cases), the Western Cape Department of Health and Wellness, and the HREC of North‒West University, Potchefstroom campus (ethics approval number: NWU-00063-18-A1-01).

## Results and discussion

### Quality assurance

A total of 21 QC samples were run at regular intervals throughout the ^1^H-NMR analysis. Quantitatively, the coefficient of variation (CV) was calculated in Excel for all spectral bins, and the bins with a QC CV greater than 10% (i.e., more than 10% analytical variation, likely due to horizontal shifting in the ^1^H-NMR spectra in regions that contain peaks that are sensitive to slight pH differences) were removed from the entire data matrix. Hence, we can assure the quality of the remaining data and that we will assess biological variances with negligible analytical variation present in the data. Furthermore, qualitatively, an evaluation of the PCA scores plot (Figure S1) showed that the QCs clustered closely together; therefore, no experimental drift of the analytical pipeline was observed in this study.

### Qualitative overview: multivariate statistics

**Fig. 2:**
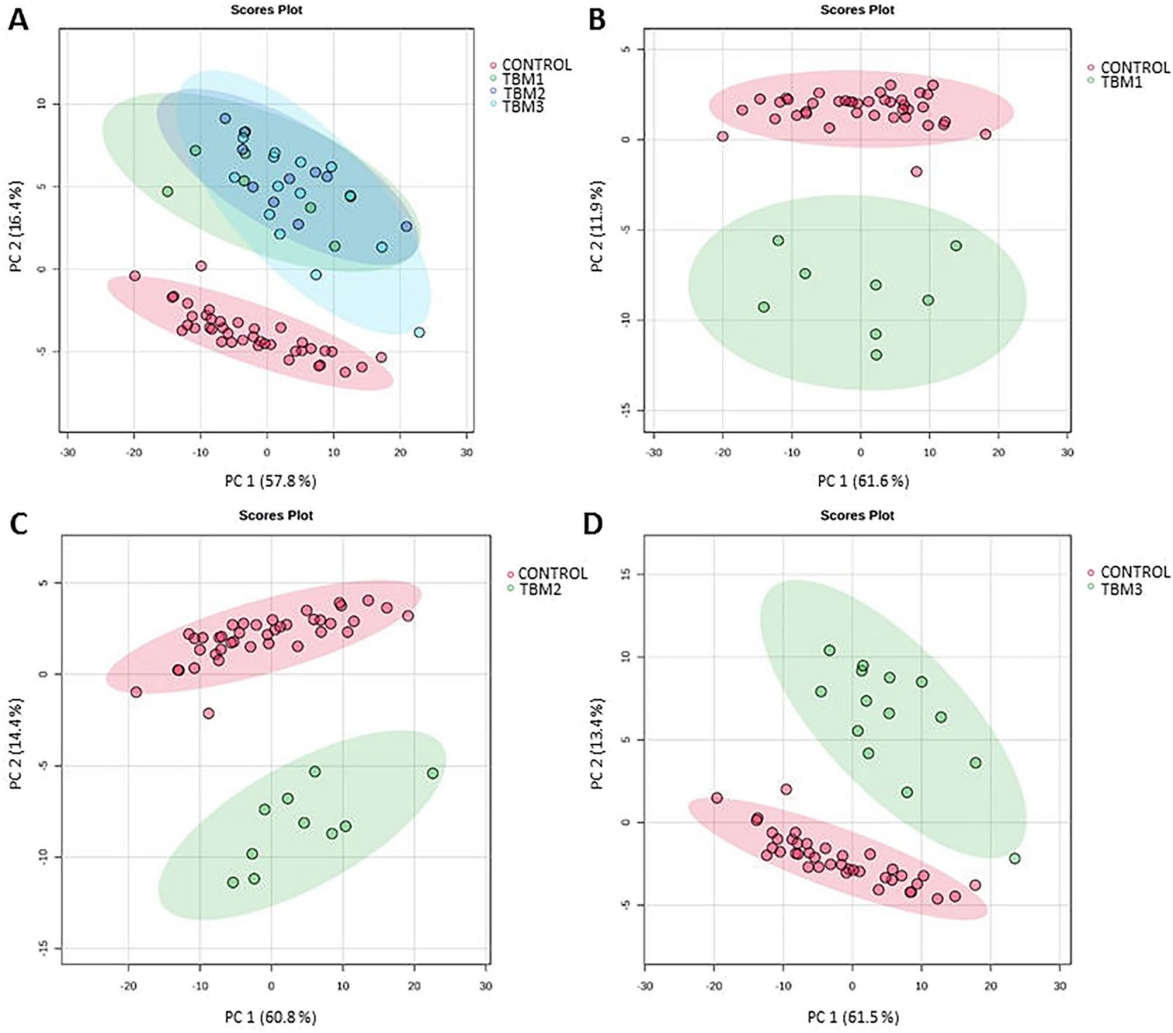
PCA score plots of (**A**) Controls vs. all three TBM stages, (**B**) Controls vs. Stage 1 TBM, (**C**) Controls vs. Stage 2 TBM, (**D**) Controls vs. Stage 3 TBM. These PCA scores plots give qualitative confirmation that natural differentiation exists between the control group and each TBM stage, supporting the use of quantitative multivariate statistical analysis.

The unsupervised PCA scores plots show natural differentiation between controls and all stages of TBM (Fig. 2A), as well as between individual comparisons (Figs. 2B-D). These qualitative results provide confidence in proceeding with additional quantitative statistical analyses—OPLSDA with RF and univariate assessment.

### Quantitative statistical evaluation

**Fig. 3:**
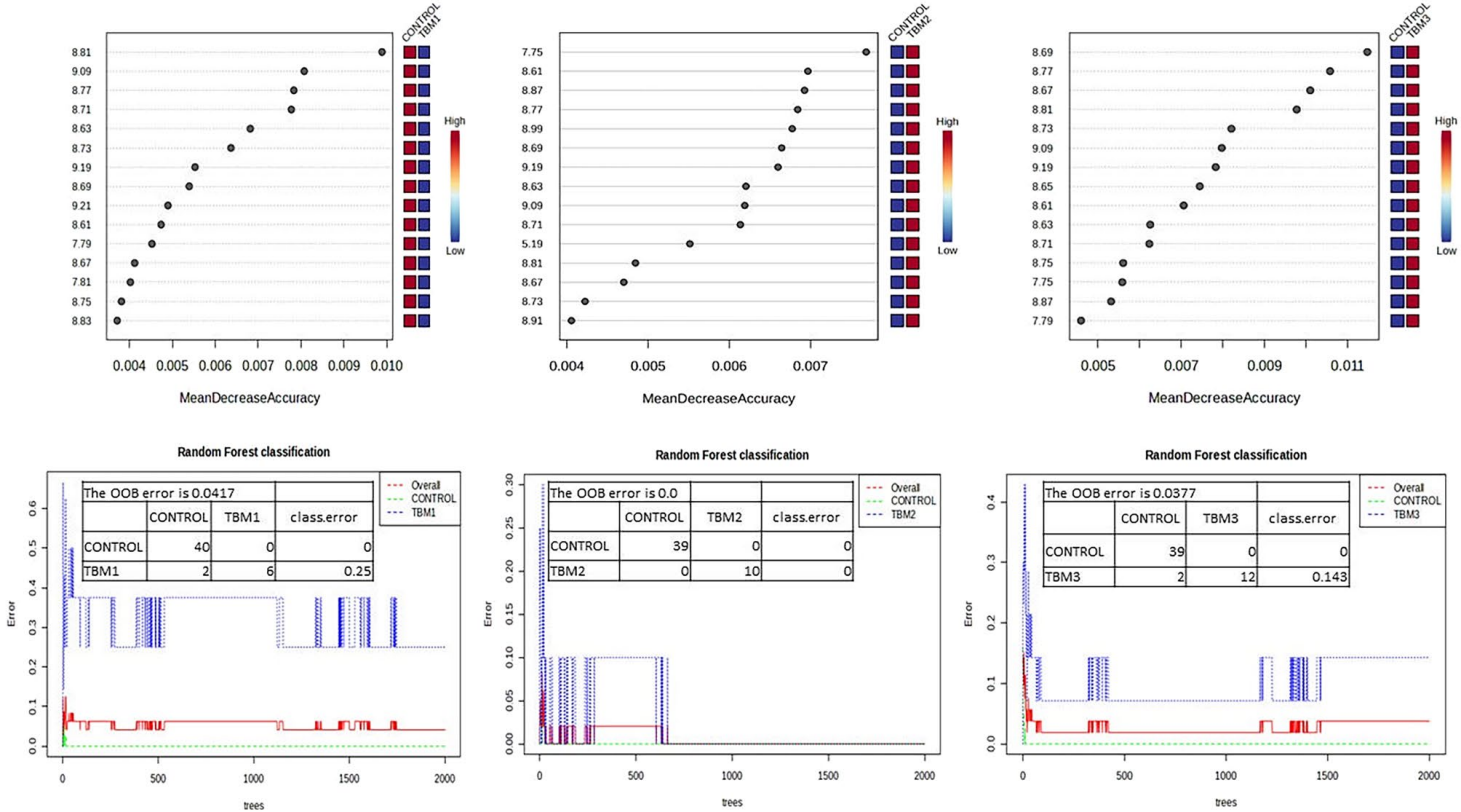
Random forest outputs supporting the OPLSDA results. The results are shown in the left, centre, and right panels for the Stage 1 TBM, Stage 2 TBM, and Stage 3 TBM, respectively. The top part of each panel shows that similar discriminating bins were found in comparison to the OPLSDA results, and the out-of-bag (OOB) values for each panel are < 0.05, supporting the OPLSDA model. The bottom part of each panel shows the Random forest classification: stage 1 TBM cases were misclassified 25% of the time as controls, and no misclassifications occurred for stage 2 TBM, while stage 3 TBM cases were misclassified 14.3% of the time as controls.

Four quantitative statistical tests, with strict critical cut-off criteria, were evaluated in our binned ^1^H-NMR data set: 1) OPLSDA with a VIP > 1.5 (supported by RF with similar discriminatory bins and OOB values < 0.05–Fig. 3), 2) Wilcoxon t test with FDR p value < 0.01, 3) fold change > 5.0, and 4) Hedge’s effect size g value > 1.0. Comparisons were made between the controls and each stage of the TBM individually. If a bin achieved at least three of the four critical cut-off criteria, then that bin was considered important for the investigation of diagnostic markers. Table 2 shows a summary of these statistically significant quantitative results. As expected, several anti-TB drugs and their metabolites were identified as significant in differentiating TBM patients on treatment from controls. Of interest to our study were the biological elements identified as significant: N-acetylglutamine, succinic acid, citric acid, 5-aminolevulinic acid, 1-methylnicotinamide, and quinolinic acid. Furthermore, 14 unknown compounds that were not found in our spectral library databases were identified as important.

**Table 2:** Quantitative statistically significant results (achieved 3 of 4 demarcated critical cut-off criteria of controls compared to TBM stages 1, 2 and 3).

Annotations	Bins	Stage 1 TBM				Stage 2 TBM				Stage 3 TBM			
		VIP	FDR	FC	ES	VIP	FDR	FC	ES	VIP	FDR	FC	ES
Rifampicin/3-formylrifampicin	0.59	2.21	< 0.01	7.65	2.27								
Rifampicin/3-formylrifampicin	0.61	2.04	< 0.01				< 0.01	8.02	2.84		< 0.01	5.50	2.06
Propylene glycol	1.13				2.26	1.56	< 0.01	35.30	3.00		< 0.01	22.53	2.11
Propylene glycol	1.15				2.84								
X1	1.55				2.70	1.52	< 0.01		3.32				
X2	1.57				4.13	1.61	< 0.01		4.79	1.52	< 0.01		3.59
N-Acetylglutamine	2.07	1.50	< 0.01			1.54	< 0.01		4.38		< 0.01	5.87	2.66
Acetylisoniazid	2.15	1.88	< 0.01		3.49	1.61	< 0.01		3.79				
Succinic acid	2.41	1.56	< 0.01		3.11	1.50	< 0.01	8.16	3.50				
5-Aminolevulinic acid	2.53	1.87	< 0.01		2.94								
Citric acid	2.67	1.51	< 0.01								< 0.01	8.01	1.62
5-Aminolevulinic acid	2.81				3.85	1.58	< 0.01		3.53	1.58	< 0.01		3.73
X3	3.17	1.73	< 0.01		3.39	1.60	< 0.01		4.26				
X4	3.39						< 0.01	7.46	1.22				
Propylene glycol	3.45				1.01								
Propylene glycol	3.47				2.53	1.56	< 0.01	5.52	2.31				
Propylene glycol	3.55	1.62	< 0.01			1.56	< 0.01		3.14	1.52	< 0.01		3.14
Propylene glycol	3.89	1.65	< 0.01				< 0.01	5.32	2.15		< 0.01	5.11	1.80
N-Acetylglutamine	4.15	1.58	< 0.01		2.16	1.63	< 0.01	8.21	2.31	1.52	< 0.01	7.59	2.35
X5	4.29				2.04	1.60	< 0.01	14.04	2.77	1.54	< 0.01	11.79	2.02
X6	4.41	1.52	< 0.01				< 0.01	5.78	1.60				
X7	4.61	1.56	< 0.01		1.96		< 0.01	5.43	1.22		< 0.01	5.87	1.30
X8	5.17				1.41		< 0.01	5.55	1.77				
X9	5.19	1.86	< 0.01				< 0.01	7.55	1.16		< 0.01	8.66	1.26
Rifampicin/3-formylrifampicin	6.33	1.62	< 0.01		3.15		< 0.01	10.10	2.08		< 0.01	9.96	2.02
Rifampicin/3-formylrifampicin	6.35	1.95	< 0.01				< 0.01	5.81	3.08		< 0.01	6.66	2.00
X10	6.81				4.40	1.57	< 0.01	23.83	4.51		< 0.01	19.22	2.81
X11	7.21				3.68		< 0.01	8.15	2.52		< 0.01	6.31	2.18
Isoniazid	7.71	1.89	< 0.01		2.19	1.50	< 0.01	28.72	1.64		< 0.01	22.71	1.22
X12	7.73	1.80	< 0.01		3.36		< 0.01	43.01	3.12		< 0.01	31.00	2.34
Isonicotinic acid	7.75	2.24	< 0.01	8.39			< 0.01	6.19	1.26		< 0.01	5.89	1.64
Isonicotinic acid	7.77	1.86	< 0.01		2.75								
2-Pyridin-4-formidoacetic acid	7.79	2.27	< 0.01	12.13	1.48						< 0.01	5.67	1.31
Acetylisoniazid	7.81	2.18	< 0.01	12.57	1.27		< 0.01	10.95	2.33		< 0.01	6.94	1.29
Quinolinic acid	8.05						< 0.01	12.68	1.10				
X13	8.59	1.78	< 0.01		1.55		< 0.01	7.40	2.21		< 0.01	7.94	1.92
Isonicotinic acid	8.61	2.34	< 0.01	21.60			< 0.01	16.45	1.00		< 0.01	8.17	1.05
Isonicotinic acid	8.63	2.38	< 0.01	44.73		1.56	< 0.01	9.51	1.22				
X14	8.65	2.02	< 0.01		1.20	1.68	< 0.01	16.13	1.65	1.62	< 0.01	16.73	1.25

The annotations given to the bins were based upon 1D and 2D ^1^H-NMR spectral library matches. *VIP* variables important in projection (OPLSDA). *FDR* Wilcoxon p value adjusted for multiple tests. *FC* fold change. *ES* Hedge’s g value effect size. X indicates an unknown compound.

### Correlations

**Fig. 4:**
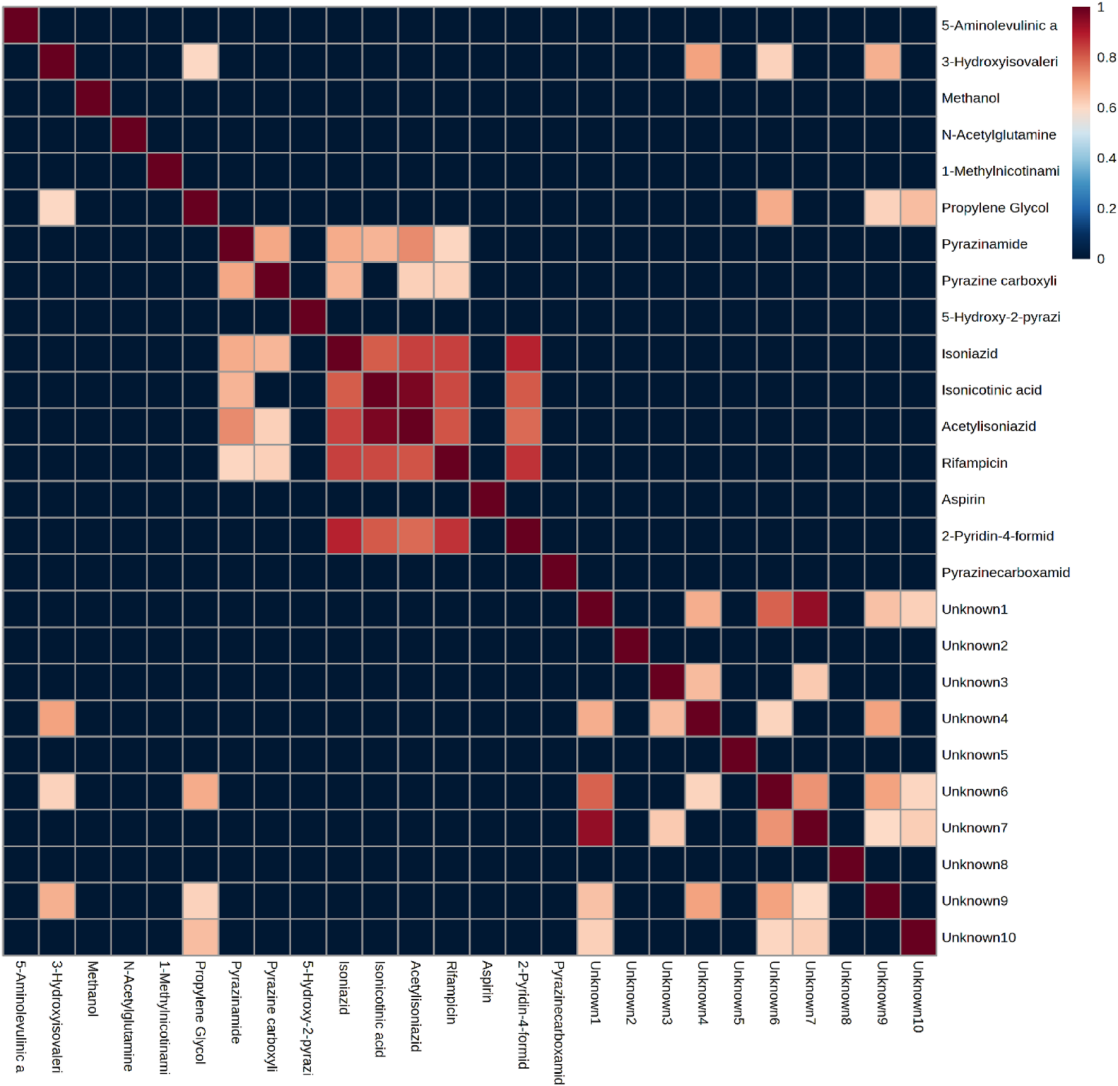
Plot showing correlations with an r value greater than 0.6 and a p value < 0.05 for all important quantified variables in Table 2 for all the TBM cases.

We also performed a correlation analysis of all the significant variables identified in Table 2 for all patients with TBM (Fig. 4). Of the five biological diagnostic metabolic markers, only 3-hydroxyisovaleric acid showed any strong correlations (r > 0.6), with statistical significance (p-value < 0.05), with other significant variables, namely, propylene glycol and unknowns 4, 6 and 9. Hence, only 3-hydroxyisovaleric acid showed some correlation with a known medication compound (propylene glycol). The other four diagnostic markers, 1-methylnicotinamide, 5-aminolevulinic acid, N-acetylglutamine and methanol, did not show strong and/or statistically significant correlations with other significant variables. These data support our argument that 1-methylnicotinamide, 5-aminolevulinic acid, and N-acetylglutamine are diagnostic markers of TBM and are not directly associated with medication.

### Evaluation of diagnostic markers for TBM

**Fig. 5:**
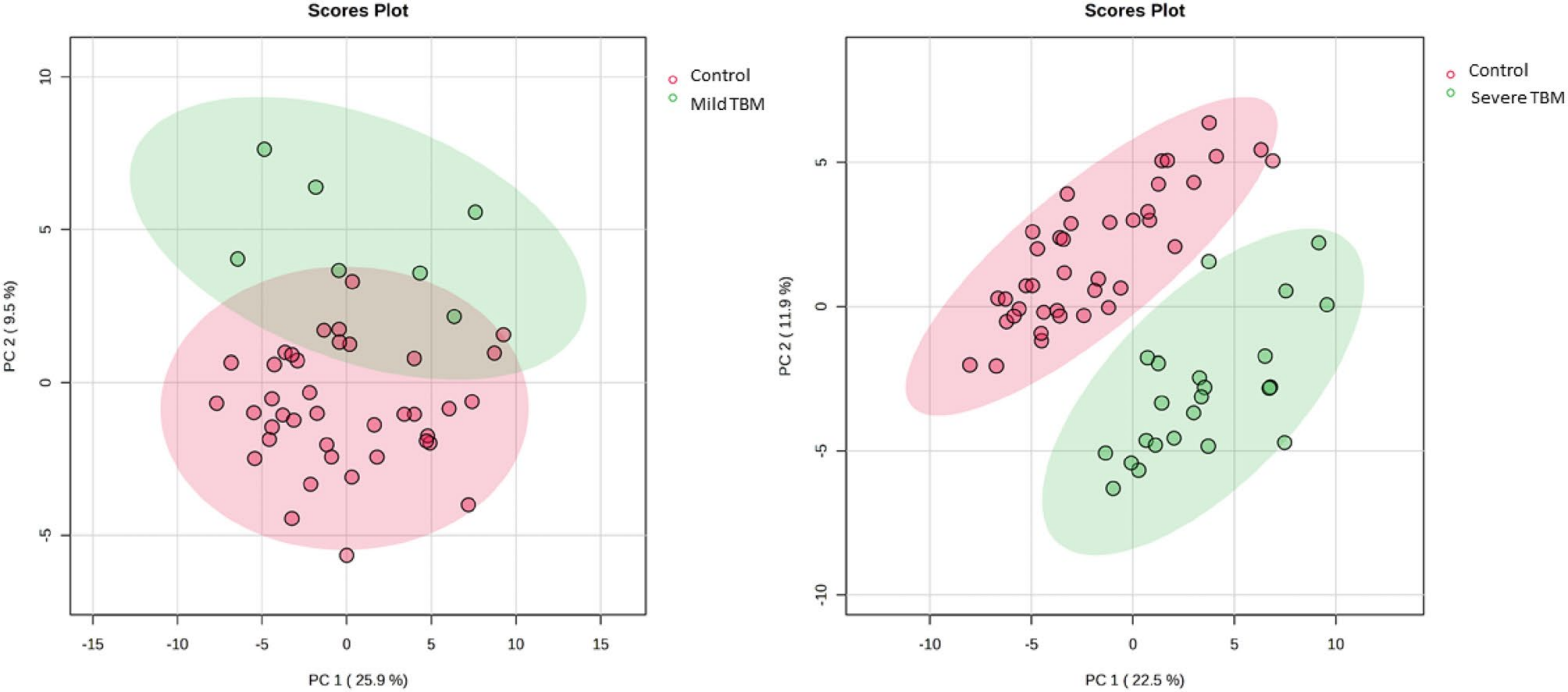
PCA score plots of revised data sets of (**LEFT**) controls vs mild TBM, showing some overlap, and (**RIGHT**) controls vs severe TBM, showing complete natural differentiation between the groups.

Since most of the significant variables annotated in Table 2 were medications and their known metabolites, we reran the statistical pipeline on a reduced data set in which all the bins related to known medications were removed. Furthermore, Fig. 2 and Table 2 show that TBM stages 2 and 3 exhibit similar characteristics. Therefore, to improve the power of the analysis, TBM stages 2 and 3 were combined and labelled ‘severe TBM’, and the remaining cases of stage 1 TBM were labelled ‘mild TBM’. Figure 5 shows the PCA score plots for this revised data set—controls vs mild TBM show some overlap, and controls vs severe TBM show complete natural differentiation. Random Forest showed that mild TBM could not be successfully classified using the current multivariate metabolic model (71.4% misclassified and OOB = 0.109). However, the random forest misclassified only one severe TBM case (4% misclassification) and had an OOB value < 0.05. Therefore, our multivariate metabolic model can successfully classify severe but not mild TBM. Furthermore, compared with the OPLSDA results, the random forest results showed similar discriminating bins.

Based on univariate statistics, using the Wilcoxon t test with an FDR p value < 0.01 and Hedge’s effect size g value > 2.0, several bins were identified as statistically and practically significant in differentiating controls from mild and severe TBM patients. Table 3 shows these quantitative statistical values, along with the annotations of the metabolites.

**Table 3:** Statistically and practically significant bins of the reduced data set based on the Wilcoxon t test with an FDR p value < 0.01 and Hedge’s effect size g value > 2.0.

Controls vs Mild TBM			Controls vs Severe TBM		
Annotations	Bins	ES	Annotations	Bins	ES
X1	1.43	2.85	3-Hydroxyisovaleric acid	1.27	2.37
N-Acetylglutamine	2.07	2.96	X2	1.55	2.59
X5	2.21	2.65	X3	1.57	2.16
X6	2.23	2.64	5-Aminolevulinic acid	2.81	2.76
5-Aminolevulinic acid	2.81	3.61	X4	2.85	2.43
X4	2.85	2.57	Methanol	3.37	2.18
X7	3.39	3.33	X7	3.39	2.55
X8	5.19	3.70	X8	5.19	2.44
X9	7.97	2.53	1-Methylnicotinamide	8.91	2.03
X10	8.33	2.09	1-Methylnicotinamide	8.99	2.47
1-Methylnicotinamide	8.99	2.59			

Annotations given to bins, based upon 1D and 2D ^1^H-NMR spectral library matches. The Wilcoxon p values, adjusted for multiple tests, was < 0.01 for all comparisons. *ES* Hedge’s g value effect size. X indicates an unknown compound.

**Fig. 6:**
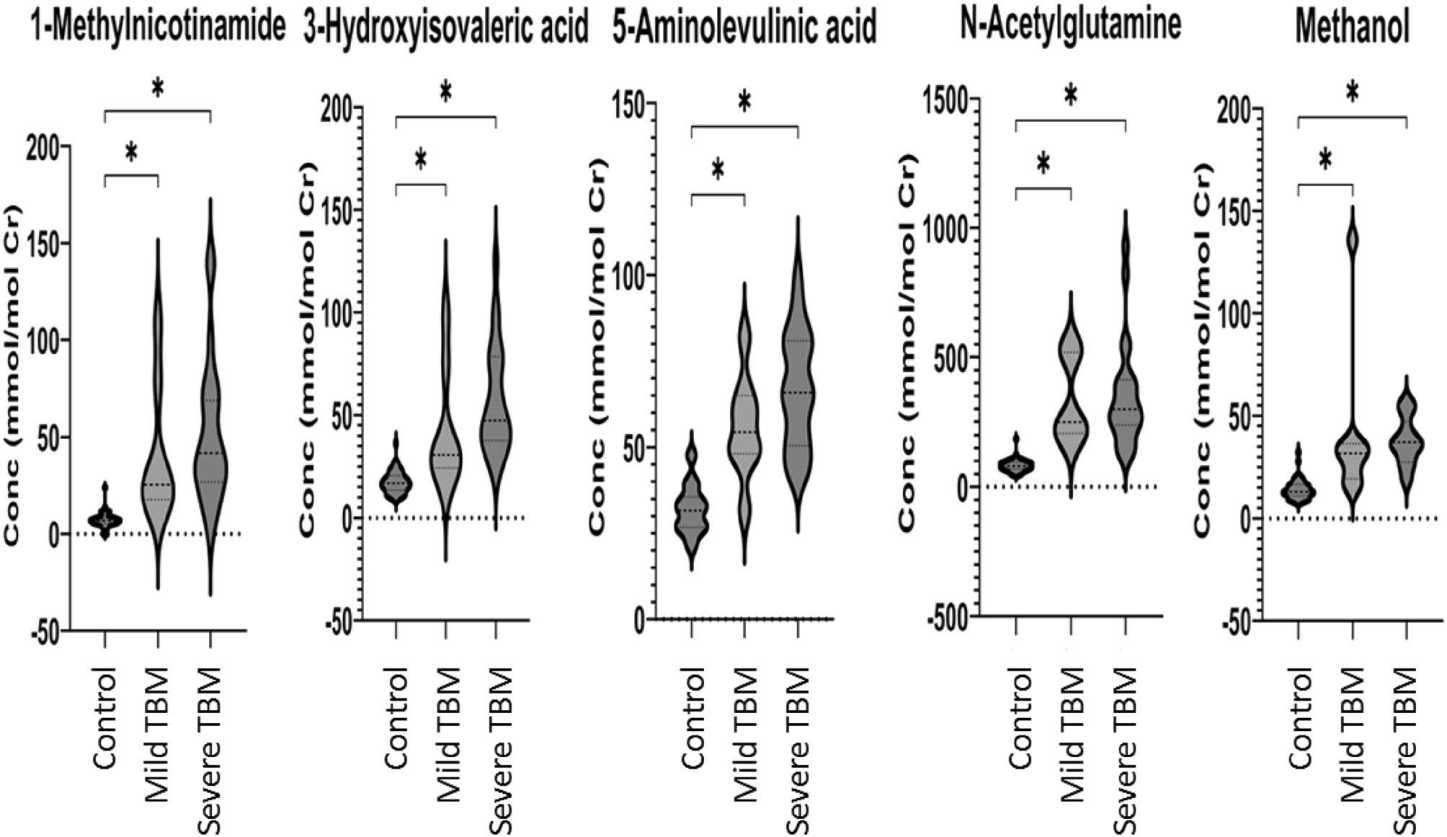
Violin plots depicting the five significant metabolites (1-methylnicotinamide, 3-hydroxyisovaleric acid, 5-aminolevulinic acid, N-acetylglutamine, and methanol) in mild and severe TBM. The concentration is given as mmol/mol creatinine in the y-axis. Statistical significance (FDR p < 0.01) is indicated by *.

**Fig. 7:**
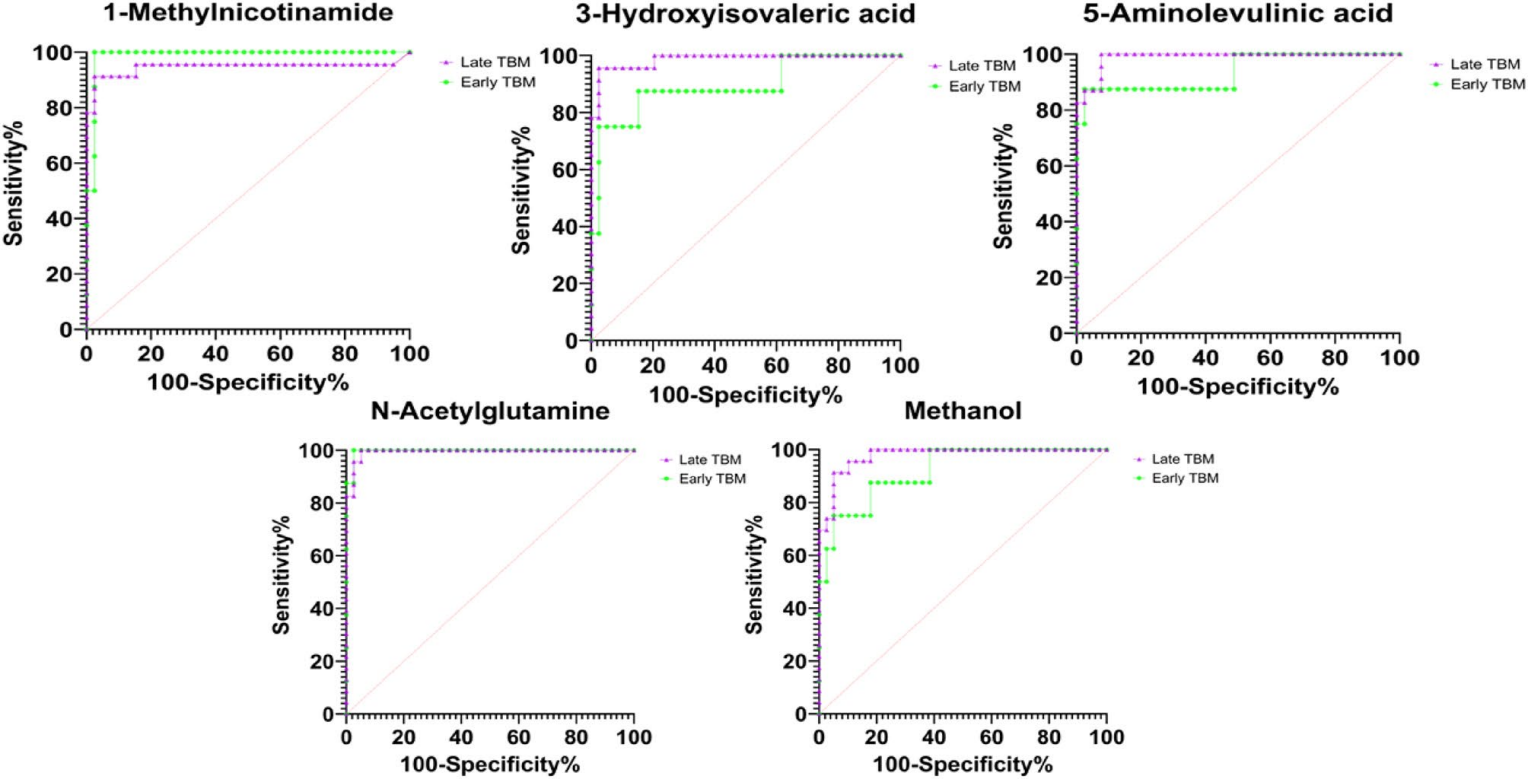
ROC curves of 1-methylnicotinamide, 3-hydroxyisovaleric acid, 5-aminolevulinic acid, N-acetylglutamine and methanol. These five ROC curves indicate that each metabolite has diagnostic potential.

Five biological metabolites from Table 3 (1-methylnicotinamide, 3-hydroxyisovaleric acid, 5-aminolevulinic acid, N-acetylglutamine and methanol) were quantified (mmol/mol creatinine); the violin plots are presented in Fig. 6. ROC curve analysis of the diagnostic potential of these five metabolites was carried out based on the area under the curve (AUC), sensitivity, and specificity (Fig. 7 and Table 4).

**Table 4:** Diagnostic sensitivity of the five urinary metabolic markers of TBM at specified cut-off concentrations.

Metabolite	Diagnostic criteria	Mild TBM	Severe TBM
1-Methylnicotinamide	Conc (mmol/mol cr)	> 14	> 14.45
	Sensitivity %	100 (67.6–100)	91.3 (73–98.5)
3-Hydroxyisovaleric acid	Conc (mmol/mol cr)	> 27.33	> 27.41
	Sensitivity %	75 (41–95.6)	95.7 (79–100)
5-Aminolevulinic acid	Conc (mmol/mol cr)	> 47.89	> 47.84
	Sensitivity %	87.5 (53–99)	87 (68–95.5)
N-Acetylglutamine	Conc (mmol/mol cr)	> 117.3	> 127.5
	Sensitivity %	100 (67.6–100)	95.7 (79–100)
Methanol	Conc (mmol/mol cr)	> 29.46	> 29.86
	Sensitivity %	62.5 (30.5–95.6)	73.9 (53.5–87.5)

The specificity for all was 97.5%, with a 95% confidence interval of 87%-100%

As shown in Fig. 7, all five metabolites have diagnostic potential. 1-Methylnicotinamide had AUCs of 0.99 and 0.95 for mild TBM and severe TBM, respectively; 3-hydroxyisovaleric acid had AUCs of 0.89 and 0.99 for mild TBM and severe TBM, respectively; 5-aminolevulinic acid had AUCs of 0.94 and 0.99 for mild TBM and severe TBM, respectively; N-acetylglutamine had AUCs of 0.99 and 0.99 for mild TBM and severe TBM, respectively; and methanol had AUCs of 0.92 and 0.98 for mild TBM and severe TBM, respectively. Table 4 shows the sensitivity of the specific cut-off concentrations, all with a specificity of 97.5% and a 95% confidence interval of 87%-100%.

1-Methylnicotinamide and N-acetylglutamine have 100% sensitivity as early diagnostic markers of mild TBM and > 90% sensitivity for severe TBM, while 3-hydroxyisovaleric acid has greater sensitivity for severe TBM, and 5-aminolevulinic acid has equivalent sensitivity as a diagnostic marker for both mild and severe TBM. Therefore, 1-methylnicotinamide, 3-hydroxyisovaleric acid, 5-aminolevulinic acid and N-acetylglutamine show potential as diagnostic markers of TBM but do not have sufficient power to differentiate mild-stage from severe-stage TBM. Methanol showed statistical significance (Fig. 6) but lacked diagnostic sensitivity for use as a diagnostic marker for TBM.

### Biological context for the four TBM diagnostic metabolites

Three (1-methylnicotinamide, 3-hydroxyisovaleric acid, and N-acetylglutamine) of the four urinary metabolic markers of TBM, as described above, have been discussed in terms of their biological context in our previous study—see^23^ for more details. Here, we report 5-aminolevulinic acid as being a significant urinary metabolite in TBM for the first time. 5-Aminolevulinic acid has been defined as a non-proteinogenic amino acid, meaning it is not used in the synthesis of proteins. This endogenous metabolite is biosynthesized in the body by the condensation of glycine and succinyl-CoA, catalysed by the enzyme 5-aminolevulinate synthase. 5-Aminolevulinic acid is required in the metabolism of heme in the body and it is involved in maintaining normal mitochondrial function.

To the best of our knowledge, this is the first time that 5-aminolevulinic acid has been identified in any type of sample matrix collected from TBM patients. However, it is important to state that 5-aminolevulinic acid is not a specific marker of TBM. 5-Aminolevulinic acid has been identified in the urine of patients following an acute attack of porphyria^32^, as well as in patients diagnosed with the tyrosinemia type I^33^. Moreover, due to its anti-inflammatory and immunoregulatory properties^34^, 5-aminolevulinic acid has been identified as a novel therapeutic for inflammatory bowel disease^35^, and has potential in treating type II diabetes mellitus^36^, and COVID-19^37^.

Hence, 5-aminolevulinic acid is likely an endogenous metabolic marker of the immune response to severe inflammation (severe and chronic neuroinflammation is a classic symptom of TBM). But, it is also important to note that some studies^38^ have identified 5-aminolevulinic acid as being neurotoxic. Therefore, future studies should examine the levels of this metabolite in the brain of TBM patients.

### TBM stage differentiation

In addition to identifying potential diagnostic markers in each TBM stage (comparing TBM patients with controls), the TBM stages were also compared to each other to determine whether any urinary metabolic marker could differentiate the TBM stages. Based on the PCA score plot (Figure S2), no qualitative differentiation could be detected between the stages of TBM (i.e., the metabolic urinary ^1^H-NMR profiles could not differentiate the stage of TBM). Quantitative statistical data were checked and did not show statistically significant differences. Therefore, no urinary metabolites could differentiate the stages of TBM.

### Unknown compounds—Biological or medication related?

Ten significant unknown compounds were identified, as shown in Table 3, and quantified. Based on the correlation data (Fig. 4), none of the unknowns were correlated with the identified anti-TB medications. However, unknowns 6, 9 and 10 were significantly correlated with propylene glycol and may be related to other forms of medication. The other unknowns (1, 2, 3, 4, 5, 7 and 8) had no correlation with medication and may be of biological origin.

### Speculation of the identities of unknowns

Based on the 2D JRES and COSY NMR spectral data, a doublet at 1.55 ppm (Unknown 2) was correlated with a quartet at 5.19 ppm (Unknown 8) (see Figure S3 in the supplementary information for more details). This ^1^H-NMR chemical shift information implies that a terminal methyl group is present next to a double bond CH component. This chemical information suggests that one of the unknowns is likely to have a chemical structure similar to that of a short-chain unsaturated carboxylic acid. The ^1^H-NMR spectra of several suspected short-chain unsaturated carboxylic acids were investigated, including propionic acid (C3), butyric acid (C4), and isobutyric acid (C4). However, while the patterns were similar, none of these pure compound spectra matched the unknown spectra sufficiently. We suspect that the unknown compound that contains a doublet at 1.55 ppm and a quartet at 5.19 ppm is a short branched-chain organic acid that is methylated. Short branched-chain organic acids originate from micro-organisms, and these unknowns unique to the TBM cases could be degradation products of the unique cell wall of *M.tb*. It is our recommendation that additional cohorts of urine samples collected from treated TBM patients should undergo targeted gas chromatography‒mass spectrometry (GC‒MS) metabolomics analyses, with a focus on short and branched-chain organic acids that are methylated.

## Conclusions

The identification of urinary biomarkers for TBM is a promising area of research. Our multivariate metabolic model can successfully classify severe but not mild TBM. The four metabolites (1-methylnicotinamide, 3-hydroxyisovaleric acid, 5-aminolevulinic acid and N-acetylglutamine) identified in this study show good diagnostic potential for severe TBM (stage 2 and 3 TBM patients combined), but they have not yet been established as definitive diagnostic tools. Future studies are needed to validate these biomarkers, including the significant unknowns identified in this study, to determine their clinical utility and explore their specificity and sensitivity for the diagnosis of TBM. Further research is needed, but we believe that urine from TBM patients will potentially aid in the earlier diagnosis and treatment of this disease.

### Supplementary Information

Supplementary Figures.

## Data Availability

All metabolomics research data associated with this project will be made available online in a data repository once the project is completed and all the data are published. On special request, immediate subsets of data from the study published here can be made accessible via email: nmr.nwu@gmail.com.
