# Supplementary material for: The diagnostic potential of urine in paediatric patients undergoing initial treatment for tuberculous meningitis
Source: Sci Rep. 2024 Aug 22;14:19471. doi: 10.1038/s41598-024-70419-1 (PMC11341861; doi:10.1038/s41598-024-70419-1)
Supplement: Supplementary file 1 — Supplementary Figures. [file 41598_2024_70419_MOESM1_ESM.docx]

**Supplementary information:**

**The diagnostic potential of urine in paediatric patients undergoing initial treatment for tuberculous meningitis**

Simon Isaiah^1^, Johan A. Westerhuis^2^, Du Toit Loots^1^, Regan Solomons^3^, Marceline Tutu van Furth^4^, Sabine van Elsland^3,5^, Martijn van der Kuip^4#^ and Shayne Mason^1#*^

^1^ Human Metabolomics, Faculty of Natural and Agricultural Sciences, North‒West University, Potchefstroom, South Africa.

^2^ Biosystems Data Analysis, Swammerdam Institute for Life Sciences, University of Amsterdam, Amsterdam, The Netherlands.

^3^ Department of Paediatrics and Child Health, Faculty of Medicine and Health Sciences, Stellenbosch University, Cape Town, South Africa.

^4^ Vrije Universiteit, Pediatric Infectious Diseases and Immunology, Amsterdam University Medical Centers, Emma Children’s Hospital, De Boelelaan 1117, Amsterdam, The Netherlands.

^5^ MRC Centre for Global Infectious Disease Analysis, Imperial College London, United Kingdom.

**Supplementary Information**


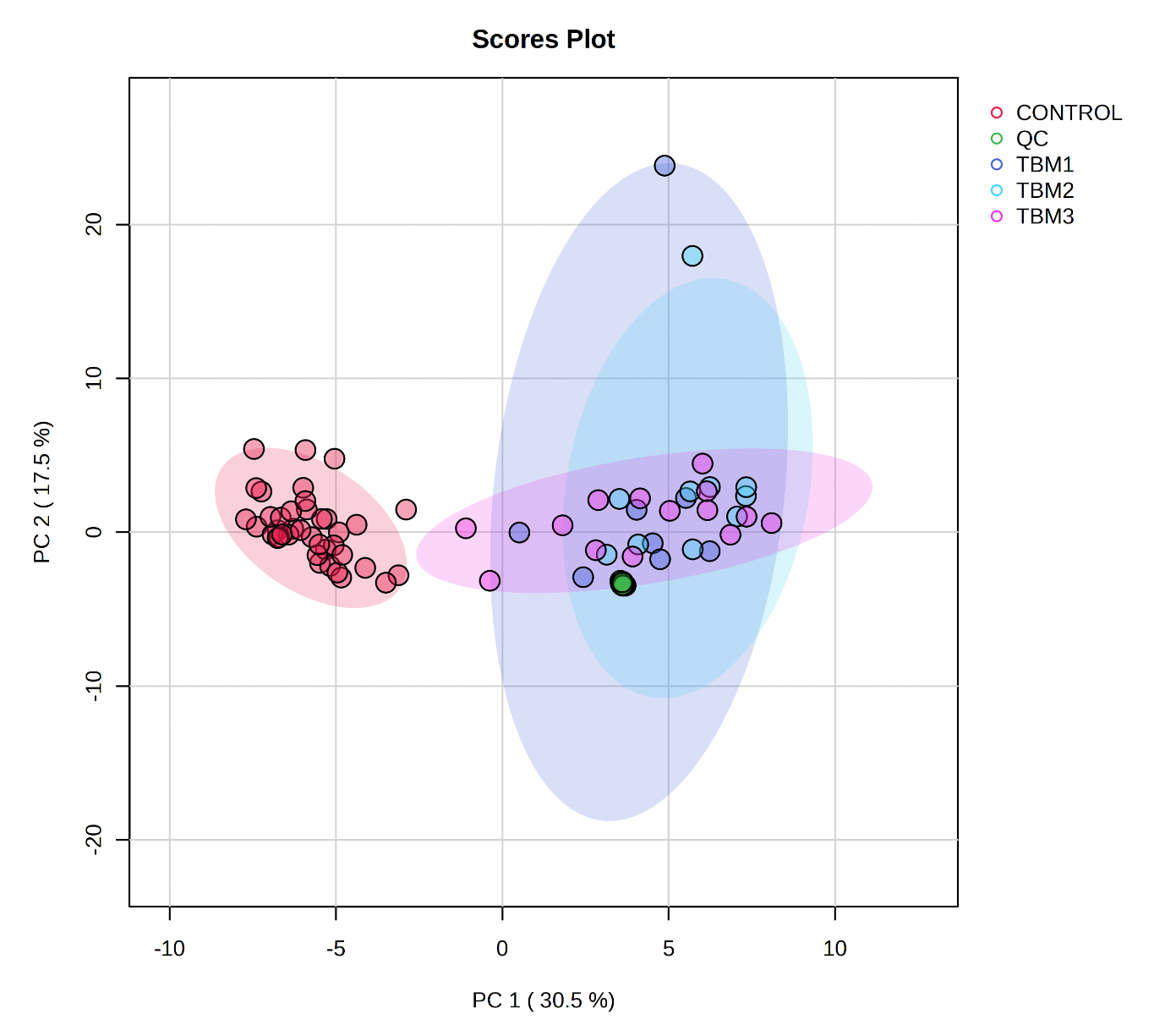


**Figure S1**: PCA scores plot of all patients and QC samples. The QC samples (green dots) clustered closely together, indicating that there was no discernible analytical drift during the course of all the ^1^H-NMR analyses.


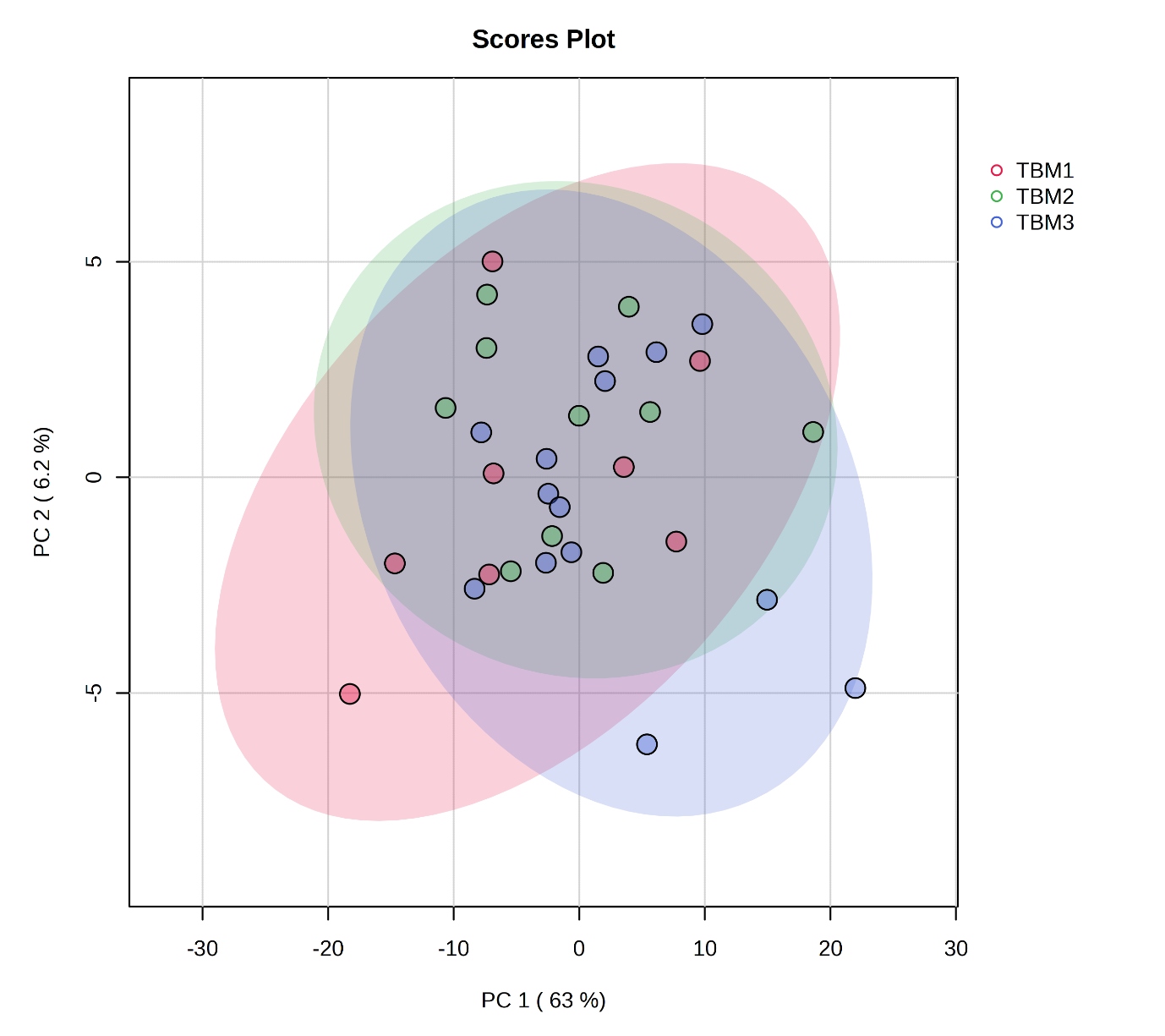


**Figure S2**: PCA scores plot of all the TBM stages showing no differentiation.


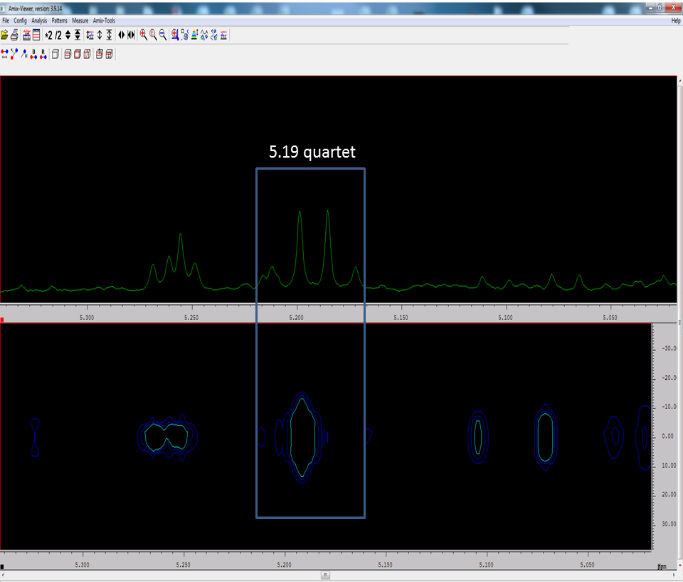

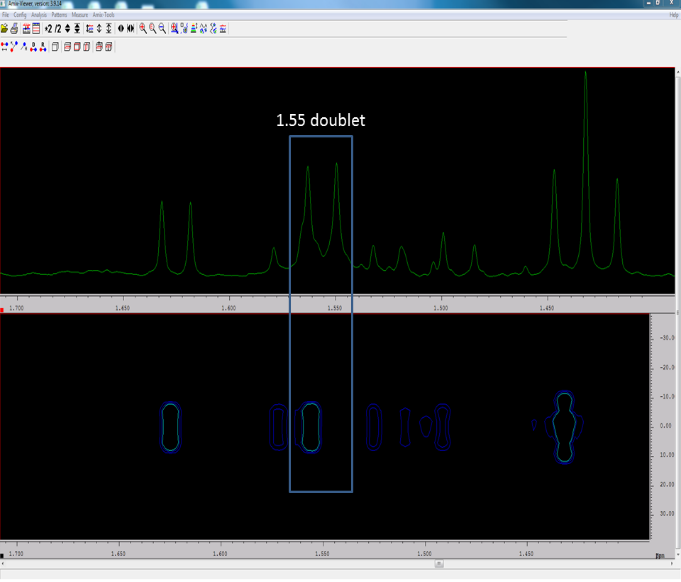


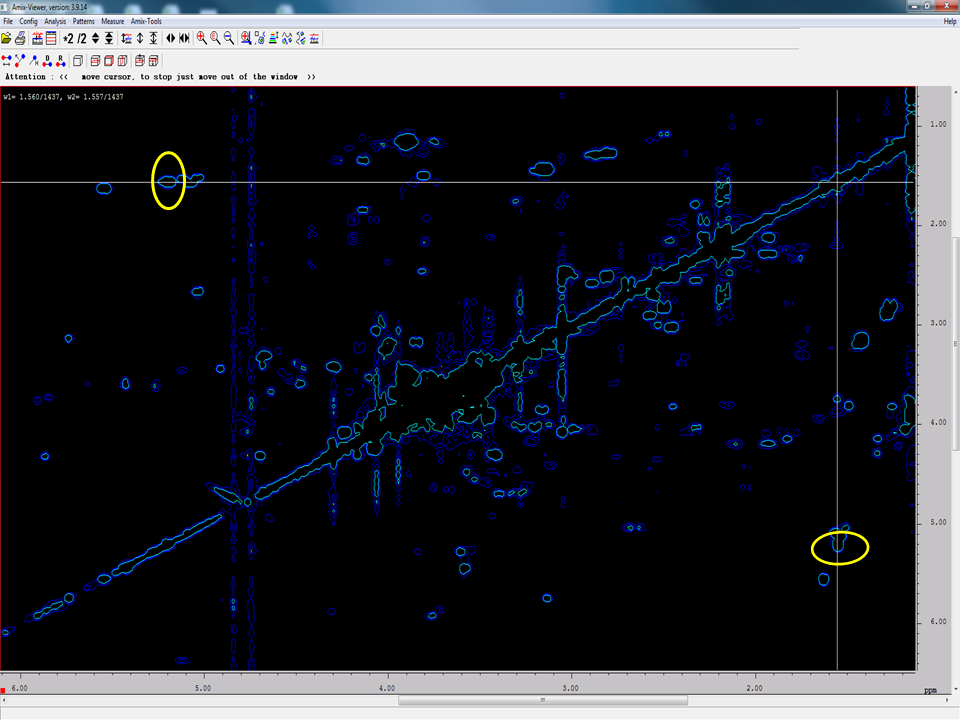


**Figure S3**: Above: 1D and 2D JRES NMR spectra showing a doublet at 1.55 ppm (above right) and quartet at 5.19 ppm (above left). Bottom 2D COSY NMR spectrum showing (yellow circles) that the doublet at 1.55 ppm and the quartet at 5.19 ppm are correlated (i.e., they are part of the same chemical compound).
